# Effect of Repeated Electroacupuncture Intervention on Hippocampal ERK and p38MAPK Signaling in Neuropathic Pain Rats

**DOI:** 10.1155/2015/641286

**Published:** 2015-06-16

**Authors:** Jun-ying Wang, Shu-ping Chen, Yong-hui Gao, Li-na Qiao, Jian-liang Zhang, Jun-ling Liu

**Affiliations:** Department of Physiology, Institute of Acupuncture and Moxibustion, China Academy of Chinese Medical Sciences, Beijing 100700, China

## Abstract

Results of our past studies showed that hippocampal muscarinic acetylcholine receptor (mAChR)-1 mRNA and differentially expressed proteins participating in MAPK signaling were involved in electroacupuncture (EA) induced cumulative analgesia in neuropathic pain rats, but the underlying intracellular mechanism remains unknown. The present study was designed to observe the effect of EA stimulation (EAS) on hippocampal extracellular signal-regulated kinases (ERK) and p38 MAPK signaling in rats with chronic constrictive injury (CCI) of the sciatic nerve, so as to reveal its related intracellular targets in pain relief. After CCI, the thermal pain thresholds of the affected hind were significantly decreased compared with the control group (*P* < 0.05). Following one and two weeks' EAS of ST 36-GB34, the pain thresholds were significantly upregulated (*P* < 0.05), and the effect of EA2W was remarkably superior to that of EA2D and EA1W (*P* < 0.05). Correspondingly, CCI-induced decreased expression levels of Ras, c-Raf, ERK1 and p-ERK1/2 proteins, and p38 MAPK mRNA and p-p38MAPK protein in the hippocampus tissues were reversed by EA2W (*P* < 0.05). The above mentioned results indicated that EA2W induced cumulative analgesic effect may be closely associated with its function in removing neuropathic pain induced suppression of intracellular ERK and p38MAPK signaling in the hippocampus.

## 1. Introduction

It has been well-documented that patients with chronic pain often experience sustained chronic psychological and physical stress and exhibit increased anxiety, depression, and deficits in working memory [1–3]. Results of a pilot study showed that in elderly patients with chronic pain, a reduced hippocampal volume and lower levels of hippocampal N-acetylaspartate to creatine ratios (NAA/Cr) were found [4]. The hippocampus, an important region of the limbic system, has been shown to be complicated in pain processing, particularly under chronic pain conditions [1–3].

Animal studies showed hippocampal abnormalities in animal models of chronic pain including short-term working memory dysfunction [5], recognition memory deficits [6], abnormal cytokine (IL-1*β* mRNA) expression [7, 8], deficits in long-term potentiation (LTP) [6], impaired enriched-environment neurogenesis [9], and altered synaptic plasticity [10]. Increasing evidence has demonstrated the involvement of hippocampus in acupuncture analgesia [11–13] and acupuncture signal processing [14, 15].

Our experimental studies demonstrated that in chronic constrictive injury- (CCI-) induced neuropathic pain rats, the resultant cumulative analgesic effect of repeated electroacupuncture stimulation (EAS) of Zusanli (ST36)-Yanglingquan (GB34) is closely associated with its effects in upregulating the decreased hippocampal synaptophysin immunoactivity [16], muscarinic acetylcholine receptor (mAChR)-1 mRNA and choline acetyl transferase (ChAT) mRNA expression [17], and improving synaptic plasticity of nerve cells in the hippocampal CA3 region shown by electron transmission microscope [18]. Differential proteomics analysis and Western blotting validation indicated that the 19 hippocampal differentially-depressed proteins involving repeated EAS-induced pain relief are those participating in metabolic, physiological, and cellular processes, and so forth, and one of the top three canonical pathways identified is “mitogen-activated protein kinase (MAPK) signaling” [19].

MAPK is an important protein molecule for intracellular signal transduction and is involved in many physiological and pathological processes of biological activity. The MAPK family mainly includes extracellular signal-regulated kinases (ERK), p38 MAPK, and c-Jun N-terminal kinase/stress-activated protein kinase (JNK/SAPK), which represent three separate signaling pathways [20, 21]. The MAPKs signaling cascades from extracellular stimuli into a variety of intracellular responses are involved in various cellular functions by sequential activation of MAPKKK, MAPKK, MAPK, and transcription factors [22]. ERK signaling pathway is a cascade involving sequential activation of Ras, Raf, mitogen-activated protein kinase (MEK), ERK, p38MAPK, MKK3, 6, and p38 (*α*, *β*, *γ*, *δ*) [20]. By using MAPK inhibitors targeting ERK, p38 MAPK, and JNK in combination with LTP recording in the dorsal hippocampus formation (HF), Liu et al. [23] demonstrated that the specific members of the MAPK family might mediate pain-associated spatial and temporal plasticity in the HF. In addition, it has been shown that after peripheral nerve injury, ERK and p38 MAPK were activated and increased in their expression levels in the spinal dorsal horns [24–26]. However, there has been no any research on the effect of repeated EAS on changes of ERK signaling pathway and p38 MAPK in the hippocampus in neuropathic pain animals. For this reason, the present study was designed to investigate the relationship between EAS-induced cumulative analgesia and activities of ERK and p38MAPK signaling in the hippocampus in CCI rats for revealing the underlying intracellular mechanism of EAS analgesic target.

## 2. Materials and Methods

### 2.1. Ethic Statement

The protocols of the present study were approved by the Institute of Acupuncture and Moxibustion, China Academy of Chinese Medical Sciences. The study was carried out in accordance with the recommendation in the Guidelines for Declaration of the National Institutes of Health Guide for Care and Use of Laboratory Animals (publication number 80-23, revised 1996). All surgical operations were performed under anesthesia, and all efforts were made to minimize animals' sufferings.

### 2.2. Animals and Grouping

Adult male Wistar rats (200–250 g), purchased from Beijing Union Medical College, were acclimatized to standard laboratory conditions (about 12 h alternate light-dark cycle) of our institute's environment first for a week and were given free access to standard chow pellet diet and water. The rats were randomly assigned to 5 groups: control, model (chronic constrictive injury, CCI), CCI + EA2D (days), CCI + EA1W (week), and CCI + EA2W, with 14 rats in each group.

### 2.3. Chronic Neuropathic Pain

The chronic pain model was established by ligature of the left sciatic nerve with reference to modified Bennett's and Xie's methods [27]. Under anesthesia (with mixture solution of urethane 28 mg/100 g plus chloralose (Sigma, 3.3 mg/100 g)) and routine sterilization, the left sciatic nerve was exposed at the midthigh level by blunt dissection through the biceps femoris muscle. Four constrictive ligatures (4–0 surgical suture) were tied around the nerve at the distal end close to the bifid site at spaces of about 1.0 mm apart. The ligature was alright till the local moderate muscular contraction of the leg could be seen clearly. After local application of antibiotic (sodium penicillin, 9,000–10,000 U/rat), the muscle and skin were sutured in layers. For rats of the control group, the left sciatic nerve was just exposed without ligature. For reducing experimental variability, all the operations were finished by the same one operator.

### 2.4. Electroacupuncture Treatment

Bilateral “Zusanli” (ST36) and “Yanglingquan” (GB34) were punctured with stainless-steel acupuncture needles (Gauge 28, 0.20 mm in diameter) to a depth of about 4 mm, respectively, and stimulated electrically by using a HANS EA Stimulator (LH202, made in China). EA (2/15 Hz, 1 mA) was given to rats for 30 min, once a day, continuously for one week (from day 12 on after CCI), 2 weeks (from day 4 on after CCI), and 2 days (from day16 on after CCI), respectively.

### 2.5. Thermal Pain Threshold Detection

When thermal hyperalgesia test was conducted, the animal was put into a black cloth bag with the hindlimbs and tail exposed to move freely. A mobile radiant heat source (a high-intensity light beam of radiant heat dolorimeter) was focused onto the plantar surface of the hindpaw. The paw withdrawal latency (PWL) (i.e., pain threshold, PT) of the rat's bilateral footplates was detected 3 times, with an interval of about 5 min between two detections. In order to avoid potential tissue damage, the cutoff time of the radiant heat radiation was set at 20 sec. The mean PT before CCI operation was used as the control value, and 4 days after CCI operation, PT was detected again. For rats of CCI + EA groups, PT was detected on the following day for observing the posteffect of EA. In order to minimize the animal individual difference, the difference value of PWL between the healthy and the affected footplates was used as the pain score.

### 2.6. Western Blot

The right hippocampus was taken to be frozen in liquid nitrogen and stored at −80°C until use. Total protein was extracted first from the tissue in RIPA Lysis Buffer containing protease and phosphatase inhibitors (Roche) by using a tissue homogenizer. The tissue lysate was then centrifuged at 13000 rpm at 4°C for 20 min, and protein concentration of supernatants was determined using a bicinchoninic acid (BCA) protein assay kit (Thermo Scientific). Equivalent amount of protein (50 *μ*g/tissue lysate) in each sample was loaded per lane and separated by 5% or 8% sodium dodecyl sulfate polyacrylamide gel electrophoresis (SDS-PAGE) for about 60 min at 90/160 V and then electrotransferred onto polyvinylidene difluoride (PVDF) membrane for 150 min at 90 m A. The membranes were blocked with 5% bovine serum albumin (BSA, Amresco, USA) solution for 30 min at room temperature. The membranes were incubated with primary antibody Ras protein (1 : 5000, Cell Signaling Technology), c-Raf protein (1 : 2000, Cell Signaling Technology), MEK1 protein (1 : 10000, abcam), P44/42 (1 : 10000, Cell Signaling Technology), P-P44/42 (1 : 5000, Cell Signaling Technology), P38 (1 : 1000, abcam), and P-P38 (1 : 2000, Epitomics) at 4°C overnight. After washing, the membranes were incubated with secondary antibody (1 : 20000 diluted with goat anti-rabbit Immunoglobulin (Ig) G or 1 : 10000 diluted with goat anti-mouse IgG) conjugated to horseradish peroxidase (Jackson Immuno Research Laboratories) for 1 h at room temperature on the following day. The membranes were developed using an enhanced chemiluminescence (ECL) detection system to transfer to film. For densitometric analyses, the blots were scanned and quantified using Total Lab Quant analysis software (TotalLab Limited, England), and the result was expressed as the ratio of target gene immunoreactivity to *β*-action immunoreactivity.

### 2.7. RNA Isolation and Quantitative Real-Time PCR

The right hippocampus samples were excised and ground into powder in liquid nitrogen. Total RNA was isolated from hippocampus with Trizol (CW0581, CWbio. Co. Ltd., Beijing, China) and then reversely transcribed using a cDNA Synthesis Kit (CW0744, CWbio. Co. Ltd., Beijing, China). The reverse-transcribed products were amplified. The primer sequences used were as follows: ERK1: forward: 5′-CGTTCAGATGTCGGTGTC-3′, reverse: 5′-AAAGGAGTCAAGAGTGGG-3′; ERK2: forward: 5′-CCAGAGTGGCTATCAAGAAG-3′, reverse: 5′-GGATGTCTCGGATGCCTA-3′; p38 MAPK: forward: 5′-GTACCTGGTGACCCATCTC-3′, reverse: 5′-GATTATGTCAGCCGAGTGTAT-3′; *β*-actin: forward: 5′-GGAGATTACTGCCCTGGCTCCTA-3′, reverse: 5′-GACTCATCGTACTCCTGCTTGCTG-3′. Quantitative real-time- (QRT-) PCR was performed in 96-well plates using the QRT-PCR detection systems (AB7500, Applied Biosystems, USA). Three different biological replicates for each sample were performed. All the cDNA samples were amplified in triplicate from the same RNA preparation and the mean value was calculated. Each reaction included 2 *μ*L of cDNA, 10 *μ*L of REALSYBR Mixture (2x), 0.8 *μ*L (10 *μ*mol/*μ*L) of both forward and reverse primers, and 7.2 *μ*L of PCR-grade water, equating to a final volume of 20 *μ*L. PCR was performed under following conditions: 10 min at 95°C, followed by 40 cycles of 15 s at 95°C, and 60 s at 60°C. Then, the fluorescence acquisition after each cycle was performed. Finally, a dissociation curve was generated by increasing temperature from 65°C to 95°C in order to verify primer specificity. All samples for each reference gene were run on the same plate to avoid between-ran variations. The relative expression was calculated in accordance with the ΔΔCT method. Relative mRNA levels were expressed as 2^−ΔΔCT^ values.

### 2.8. Statistical Analysis

The data collected in the present study were presented as mean ± standard deviation (mean ± SD) and analyzed by two-way repeated measures ANOVA, followed by* post hoc *test for least significant difference (LSD) to determine differences between every two groups. Statistical significance was accepted with *P* < 0.05.

## 3. Results

### 3.1. Effect of EA on Pain Response after CCI

**Figure 1:**
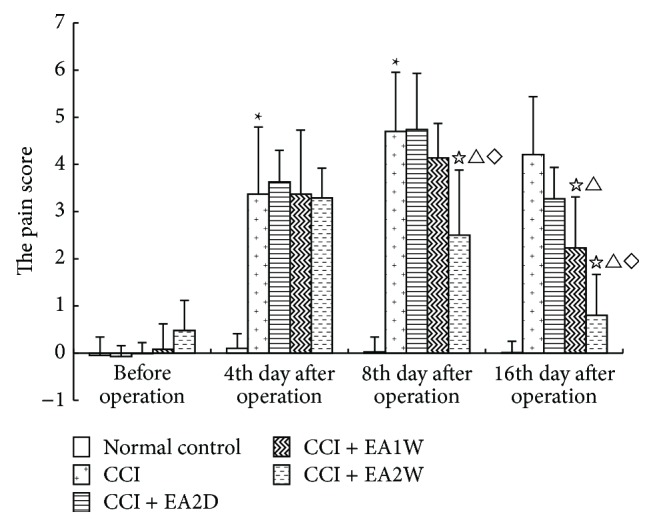
Effect of EA of ST36-GB34 on pain scores in CCI rats of different groups. Thermal pain thresholds after injury and EA are presented as mean ± SD (*n* = 11 in each group; ^*∗*^ *P* < 0.05 compared with the sham control group; ^☆^ *P* < 0.05, compared with the CCI group; ^△^ *P* < 0.05, compared with the CCI + EA2D group; ^⋄^ *P* < 0.05, compared with the CCI + EA1W group). Pain score = the paw withdrawal latency (PWL) of the healthy side (right) – PWL of the affected side (left).

The pain score is referred to the paw withdrawal latency of the difference between the healthy and the surgical footplates in the present paper. Results (Figure 1) indicated that before CCI, the pain scores of the control (sham operation), CCI model, CCI + EA2D, CCI + EA1W, and CCI + EA2W groups had no significant difference (*P* > 0.05). After CCI, the pain scores of the CCI group were evidently higher than those of the control group (*P* < 0.05), suggesting a hyperalgesia after CCI. On day 4 after CCI, the pain scores of the model group and those of the CCI + EA2D and CCI + EA1W and CCI + EA2W groups were comparable (*P* > 0.05), while on day 8, the pain scores of the CCI + EA2W group and, on day 20, those of the CCI+EA1W and CCI + EA2W were obviously lower than those of the model group (*P* < 0.05), and the effect of the CCI + EA2W group was significantly better than that of the CCI + EA2D and CCI + EA1W groups (*P* < 0.05), suggesting a cumulative analgesic effect of repeated EAS of ST36-GB34.

### 3.2. Effect of EA on Expression of Hippocampal Ras and C-Raf Protein in Different Groups

Ras is a membrane-associated guanine nucleotide-binding protein that is normally activated in response to the binding of extracellular signals [28], and the Raf kinase mediates the transduction of proliferative and differentiative signals from a variety of cell surface receptors to the nucleus and is the entry point to the MAPK/ERK-1/2 signaling pathway, which controls fundamental cellular functions [29].

**Figure 2:**
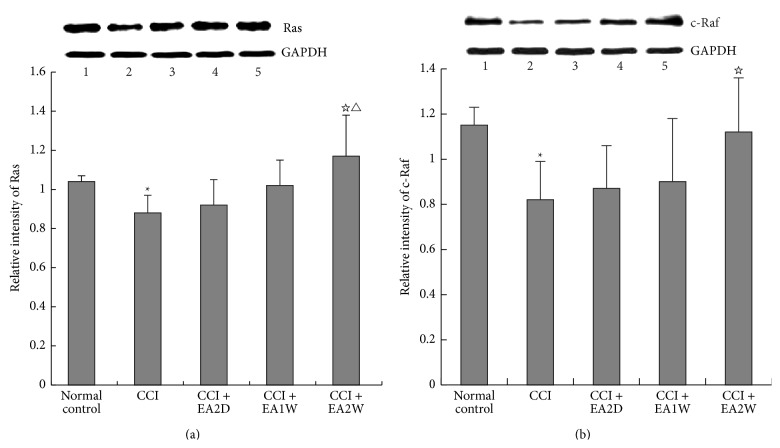
Effect of EAS on expression levels of hippocampal Ras and c-Raf proteins in different groups. After EA treatment, hippocampus tissues were prepared for assaying expression levels of Ras, c-Raf, and other related kinases (MEK, ERK, p38 MAPK) by Western blot. Data are presented as mean ± SD (^*∗*^ *P* < 0.05, compared with the sham control group; ^☆^ *P* < 0.05, compared with the CCI group; ^△^ *P* < 0.05, compared with the CCI + EA2D group; *n* = 5 in each group). (a) Top panel shows immunoblots of Ras and c-Raf proteins and GAPDH in different groups: (1) sham control group, (2) CCI group, (3) CCI + EA2D group, (4) CCI + EA1W group, and (5) CCI + EA2W group. GAPDH: glyceraldehyde-3-phosphate dehydrogenase (housekeeping gene); (b) histograms show the relative expression of Ras and c-Raf proteins in the 5 groups.

Following CCI, hippocampal Ras and c-Raf protein expression levels were significantly downregulated in comparison with those of the control group (*P* < 0.05, Figures 2(a) and 2(b)). After EAS of ST36 and GB34, both Ras and c-Raf expression levels were considerably upregulated only in the CCI + EA2W group (*P* < 0.05), rather than in the CCI + EA2D and CCI + EA1W groups (*P* > 0.05) in spite of mild upregulation. The effect of the CCI + EA2W group in upregulating Ras protein was significantly better than that of the CCI + EA2D and CCI + EA1W groups (*P* < 0.05). No significant difference was found between the CCI + EA2D and CCI + EA1W groups (*P* > 0.05).

### 3.3. Effect of EA on Hippocampal MEK and p-MEK1/2 Protein Expression in Different Groups

**Figure 3:**
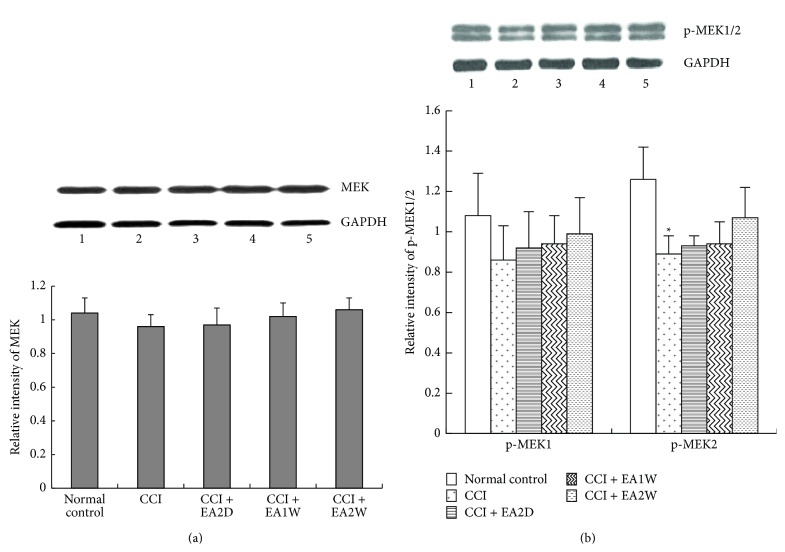
Effect of EA on expression levels of hippocampal MEK, p-MEK proteins in different groups. Data are presented as mean ± SD (^*∗*^ *P* < 0.05, compared with the sham control group; *n* = 5 in each group). (a) Upper panel shows representative immunoblots of MEK protein in the 5 groups: (1) sham control group, (2) CCI group, (3) CCI + EA2D group, (4) CCI + EA1W group, and (5) CCI + EA2W group; lower histograms show the relative expression levels of MEK protein in the 5 groups. (b) The lower histograms show the relative expression levels of p-MEK1 and p-MEK2 proteins in the five groups; upper panel shows the representative immunoblots of MEK1/2 proteins and GAPDH in different groups.

MEK1/2 (MKK1/2) are the upstream kinases of ERK signaling. Compared with the control group, the expression levels of hippocampal MEK and p-MEK1 proteins had no significant changes in the CCI, CCI + EA2D, CCI + EA1W, and CCI + EA2W groups (*P* > 0.05, Figure 3(a)), while that of p-MEK2 protein was significantly downregulated after CCI (*P* < 0.05, Figure 3(b)). Following EAS of ST36-GB34, p-MEK2 expression had a slight upregulation in the three EAS groups (*P* > 0.05) without significant differences among the three groups (*P* > 0.05).

### 3.4. Effect of EA on Hippocampal ERK and p-ERK mRNA and Protein Expression

**Figure 4:**
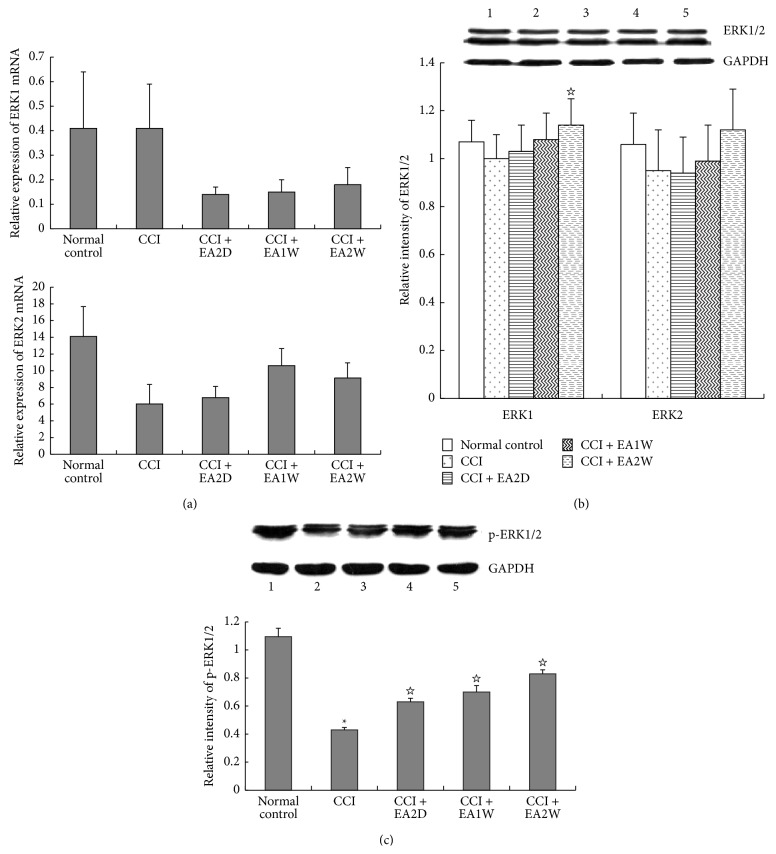
Effect of EA on expression levels of hippocampal ERK1/2mRNA and ERK1/2 and p- ERK1/2 protein in different groups. Hippocampal ERK1/2mRNA expression levels were assessed by real-time PCR and ERK1/2 protein expressions were detected by Western blot. Data are presented as mean ± SD (^*∗*^ *P* < 0.05, compared with the sham control group; ^☆^ *P* < 0.05, compared with the CCI group; *n* = 6 in each group for real-time PCR; *n* = 5 for each group for western blot); (a) histograms show the expression levels of ERK1/2 mRNA. (b) The top panel shows the representative immunoblots of ERK1/2 proteins in the 5 groups: (1) sham control group, (2) CCI group, (3) CCI + EA2D group, (4) CCI + EA1W group, and (5) CCI + EA2W group. The histograms show relative expression levels of ERK1/2 proteins in the 5 groups. (c) The upper panel shows the representative immunoblots of p-ERK1/2 proteins in the 5 groups. The lower bar graph shows the relative expression of p-ERK1/2 proteins in the 5 groups.

Like MEK, ERK exists in two isoforms (1 and 2). In order to identify changes of hippocampal ERK1/2 in both mRNA and protein expression levels, we conducted real-time PCR and Western blot measurements. Compared with the control group, the expression levels of hippocampal ERK1/2mRNA and ERK1/2 protein in the CCI group had no significant changes (*P* > 0.05), except for a marked upregulation of ERK1 protein expression in the CCI + EA2W group in comparison with the CCI group (*P* < 0.05, Figures 4(a) and 4(b)). Further tests revealed that the relative expression of p-ERK1/2 protein was considerably downregulated in the CCI group compared with the control group (*P* < 0.05, Figure 4(c)) and obviously upregulated in the CCI + EA2D, CCI + EA1W, and CCI + EA2W groups after EAS (*P* < 0.05). There was no significant difference among the three EAS groups in hippocampal p-ERK1/2 protein expression levels (*P* > 0.05, Figure 4(c)).

### 3.5. Effect of EA on Hippocampal p38 MAPK mRNA and Protein Expression

**Figure 5:**
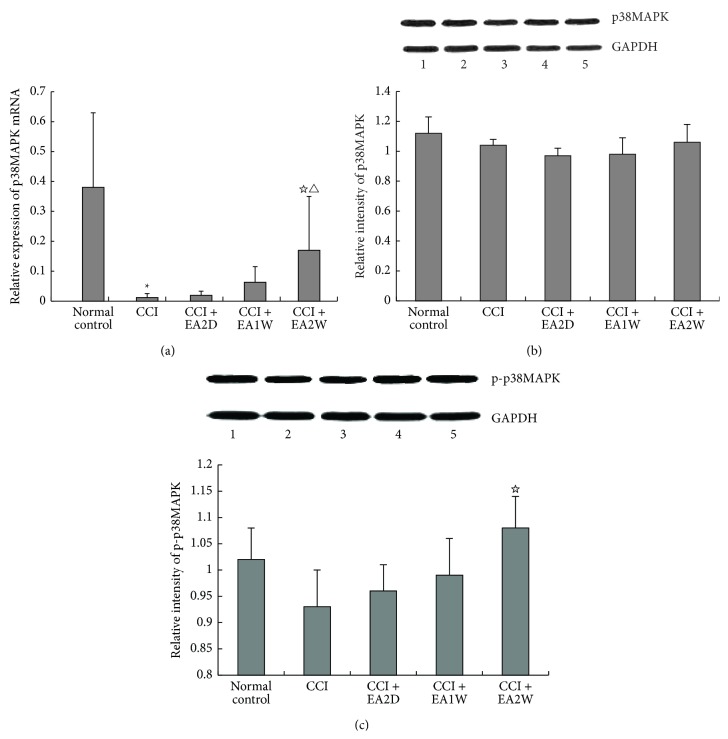
Effect of EA on expression levels of hippocampal p38MAPKmRNA and p38MAPK and p-p38MAPK proteins in different groups. Hippocampal p38MAPK mRNA and p-38MAPK and p-p38MAPK protein expression levels were assayed by real-time PCR and Western blot, respectively. Data are presented as mean ± SD (^*∗*^ *P* < 0.05, compared with the sham control group; ^☆^ *P* < 0.05, compared with the CCI group; ^△^ *P* < 0.05, compared with the CCI + EA2D group; *n* = 6 in each group for real-time PCR; *n* = 5 for each group for Western blot). (a) Histograms of real-time PCR show the expression levels of p38MAPKmRNA in the 5 groups; (b) the top panel shows the representative immunoblots of p38MAPK protein and GAPDH in (1) sham control group, (2) CCI group, (3) CCI + EA2D group, (4) CCI + EA1W group, and (5) CCI + EA2W group. The histograms show the relative expression levels of p38MAPK protein in the 5 groups. (c) Upper panel shows the representative immunoblots of p-p38MAPK in (1) normal control group, (2) CCI group, (3) CCI + EA2D group, (4) CCI + EA1W group, and (5) CCI + EA2W group. The histograms show the relative expression levels of p-p38MAPK protein in the 5 groups.

Activation of MAPK is the final step of intracellular phosphorylation cascade reactions in response to extracellular signal. Compared with the control group, hippocampal p38MAPKmRNA and p-P38MAPK protein expressions were significantly and moderately downregulated, respectively, in the CCI group (*P* < 0.05, Figures 5(a) and 5(c)). Following EA of ST36-GB34, both p38MAPKmRNA and p-P38MAPK protein were obviously upregulated only in the CCI + EA2W group (*P* < 0.05). There were no significant changes of hippocampal p38MAPK protein expression in the five groups and p38MAPK mRNA and p-p38MAPK protein expression in the CCI + EA2D and CCI + EA1W groups (*P* > 0.05, Figures 5(a), 5(b), and 5(c)).

## 4. Discussion

Results of the present study showed that following CCI, the pain threshold of the affected paw was significantly lowered and the difference values of PWL of the bilateral paws (pain scores) were apparently increased, peaking on day 8 after CCI, which is similar to Bennett's and Xie's outcomes [27]. Following EAS of ST36-GB34, the pain threshold was markedly increased in both EA1W and EA2W groups, but not in the EA2D group, presenting a cumulated analgesic effect after repeated EAS, which are identical to our results of past studies [17, 30, 31] and related reports [32, 33].

Correspondingly, after CCI, the expression levels of intracellular Ras, c-Raf, p-MEK proteins, ERK2 mRNA, p-ERK1/2 protein, and p38MAPK mRNA were obviously downregulated and that of p-p38MAPK protein was moderately downregulated in spite of the fact that there was no statistical significance. It suggests an inhibition of hippocampal ERK/MAPK signaling after CCI in neuropathic pain rats. These results of hippocampal molecules are also basically identical to those of Mutso et al. report [10] which showed reduced ERK expression and phosphorylation in the hippocampus in spared nerve injury (SNI) (tight ligature and severing of the tibial and common peroneal nerves) mice and to Liu and colleagues' study [23] about an involvement of ERK and p38MAPK in pain processing in the dorsal hippocampus formation, in which ERK and p38 MAPK seemed to play opposing roles, with the former positively involved and the latter negatively involved. CCI may be considered to be chronic stress stimulation and chronic pain often resulting in depression. Thus, some molecular changes of the hippocampus under chronic stress and depression conditions may also be used as references. It was reported that chronic stress exposure caused a reduction in p-ERK and p-CREB expression in the hippocampus of rats [34, 35]. In terms of depression caused by chronic pain [1, 10, 36–38], it was demonstrated that chronic unpredictable stress (CUS) suppressed p-ERK, p-ERK1/2, and p-CREB expression in the hippocampus.

On the other hand, controversial results do exist; for instance, it was reported that 14 days of stress induced an increase in p-ERK1/2 and p-CREB expression in the hippocampus in rats with infraorbital nerve injury [39]. Under acute conditions, Guo et al. [22] observed that in the hippocampus of naïve rats, intraplantar saline or bee venom injection mimicking transient or persistent pain equally initiated an intense and long-lasting activation of hippocampal ERKs and ERK1 which were more remarkably activated than ERK2 in the hippocampus. The possible explanations for the discrepancy may lie in the difference in stress category, duration, and other experimental procedures, and the acute pain is quite different from chronic pain in the underlying mechanisms. Moreover, in the dorsal horns of the spinal cord, the ERK signaling pathway plays an important role in the genesis and maintenance of pain, which exhibited upregulation of the expression of ERK and phosphorylated ERK proteins under peripheral nerve and tissue injury conditions [27, 40, 41].

Just as those mentioned above, p38 MAPK, an important member of the MAPKs, plays an important role in the development of central sensitization in responding to chronic nociceptive stimulation shown at the spinal cord level. Following peripheral nerve injury, p38MAPK and ERK were activated in spinal microglia, and JNK was activated in astrocytes [24, 42]. However, in the hippocampus, there has been no direct evidence for its involvement in pain processing. In view of chronic neuropathic pain induced complications as persistent stress, depression, deficits of memory, and abnormal neural plasticity changes, some findings may be used as reference evidence supporting our results. For example, as a mediator of cellular stresses, p38MAPK was implicated in depression induced by forced swim tests and tail suspension tests, exhibiting an intensive phosphorylation of PKC-dependent ERK1, ERK2, JNK, and p38MAPK in the hippocampus [43]. However, in CUS rats with impaired spatial memory, significantly decreased p-CREB and pJNK levels, but without statistical changes in CREB, ERK1/2, p-ERK1/2, p38MAPK, p-P38MAPK, and JNK levels, were found in the hippocampus [44].

Regarding the effect of EAS of bilateral ST36-GB34 on hippocampal ERK and p38MAPK signaling in the present study, following two weeks' EAS, along with the appearance of cumulative analgesia, the CCI-induced decreased expression levels of Ras, c-Raf, ERK1, p-ERK1/2 proteins, and p38 MAPK mRNA and p-pMAPK protein were considerably and gradually upregulated in the hippocampus, denoting a normalizing trend of functional activities of nerve cells under EAS-induced pain relief conditions. Most of those proteins were upregulated but had no significant changes after 2 days and one week's EAS, suggesting that two weeks' EAS has a cumulative effect in upregulating the activities of ERK and p-38 MAPK signaling along with the appearance of cumulative analgesic effect. These results are also consistent with our past results about expression levels of cellular membrane receptors including mAChR1 mRNA and protein [17] and presynaptic synaptophysin [16] in which two weeks' EAS evidently suppressed CCI-induced decrease of their expression in CCI rats. These results indicate that the EAS targets multiple signal transmission sites from extracellular to intracellular events during cumulative analgesia induction, and intracellular ERK and p-38 MAPK signal pathways play an important role in this pain processing. As we know that mAChRs are attributed to G protein-coupled receptors (GPCRs) which are critical players in converting extracellular stimuli into intracellular signals in response to various signaling inputs, and these signal inputs have to be integrated for the processing of complex biological responses. Chan et al. proved that G protein signals can be integrated at the level of MAPK, resulting in differential effects on ERK, JNK, and p38 MAPK in human brain neuroepithelioma cells as a neuronal model [45]. Despite a great variety of components of the MAPK/ERK signaling cascade, the architecture of the signal pathway is usually known as the Ras-Raf-MEK-ERK pathway [46]. Combining our past partial research results, a complete network linking the presynaptic synaptophysin, mAChR, and Ras-Raf-MEK-ERK pathway and synaptic remodeling [18] may participate in the cumulative analgesic effect of EAS in neuropathic pain.

There have been no similar reports available about the effect of EAS on hippocampal ERK and MAPK signaling in neuropathic pain animal models up to now. Therefore, we have no way to compare our outcomes with others' outcomes. However, some results may be used as a reference. For example, in depression model rats, EA could reverse CUS induced considerable upregulation of p-ERK expression, ratio of p-ERK1/2 to ERK1/2 and the ratio of p-CREB to CREB in the hippocampus [47], or enhanced the activation of hippocampal ERK signaling pathway [48], suggesting an involvement of hippocampal ERK–CREB signaling in EAS-induced antidepressant-like effects. At the spinal level, EAS could suppress complete Freund's adjuvant- (CFA-) induced activation or phosphorylation of p38MAPK in rats with inflammatory pain [49, 50]. In contusion injury induced below-level neuropathic pain rats, acupuncture stimulation of Shuigou (GV26) and Yanglingquan (GB34) relieved mechanical allodynia and thermal hyperalgesia and simultaneously inhibited neuropathic pain induced activation of p38MAPK and ERK in microglia at the L4-5 spinal cord. Injection of p38MAPK or ERK inhibitors attenuated neuropathic pain [51]. These results denote that intracellular ERK and p-38 MAPK signaling pathways in the central nervous system are involved in nociceptive information processing in chronic pain model animals.

## 5. Conclusion

In conclusion, results of the present study once again demonstrated the cumulative analgesic effect of repeated EAS of ST36-GB34 in CCI-induced neuropathic pain rats and reduce CCI-induced downregulation of Ras, c-Raf, ERK1, p-ERK1/2 proteins, and p38 MAPK mRNA and p-pMAPK protein in the hippocampus, suggesting an involvement of both ERK and p38 MAPK signaling of hippocampal nerve cells in EAS-induced pain relief. It is sure that this conclusion should be further confirmed by other approaches in the future.
